# Multiparametric magnetic resonance imaging can exclude prostate cancer progression in patients on active surveillance: a retrospective cohort study

**DOI:** 10.1007/s00330-020-06997-1

**Published:** 2020-06-26

**Authors:** T. Ullrich, C. Arsov, M. Quentin, F. Mones, A. C. Westphalen, D. Mally, A. Hiester, P. Albers, G. Antoch, L. Schimmöller

**Affiliations:** 1 Medical Faculty, Department of Diagnostic and Interventional Radiology, University Dusseldorf, 40225 Dusseldorf, Germany; 2 Department of Radiology and Biomedical Imaging, University of California, San Francisco, 505 Parnassus Ave, M-392, San Francisco, CA 94143-0628 USA; 3 Medical Faculty, Department of Urology, University Dusseldorf, Moorenstr. 5, 40225 Dusseldorf, Germany

**Keywords:** Magnetic resonance imaging, Prostate cancer, Early diagnosis, Imaging-guided biopsy, Assessment, risk

## Abstract

**Objectives:**

To assess the ability of multiparametric MRI (mp-MRI) of the prostate to exclude prostate cancer (PCa) progression during monitoring patients on active surveillance (AS).

**Methods:**

One hundred forty-seven consecutive patients on AS with mp-MRI (T2WI, DWI, DCE-MRI) at 3T were initially enrolled. Fifty-five received follow-up mp-MRI after a minimum interval of 12 months and subsequent targeted MR/US fusion-guided biopsy (FUS-GB) plus concurrent systematic transrectal ultrasound-guided (TRUS-GB) biopsy as reference standard. Primary endpoint was the negative predictive value (NPV) of the follow-up mp-MRI to exclude histopathologic tumor progression using PRECISE recommendations. Secondary endpoints were the positive predictive value (PPV), sensitivity, specificity, Gleason score (GS) upgrades, and comparison of biopsy method.

**Results:**

Of 55 patients, 29 (53%) had a GS upgrade on re-biopsy. All 29 patients showed a tumor progression on follow-up mp-MRI. Fifteen of 55 patients (27%) displayed signs of tumor progression, but had stable GS on re-biopsy. None of the 11 patients (20%) without signs of progression on follow-up mp-MRI had a GS upgrade on re-biopsy. The NPV was 100%, PPV was 66%, sensitivity was 100%, and specificity 42%. FUS-GB resulted in GS upgrade significantly more often (*n* = 28; 51%) compared with TRUS-GB (*n* = 12; 22%; *p* < 0.001).

**Conclusions:**

(Follow-up) Mp-MRI can reliably exclude PCa progression in patients on AS. Standard serial re-biopsies might be waived if follow-up mp-MRIs are stable. Over 60% of patients with signs of tumor progression on mp-MRI during AS had a GS upgrade on re-biopsy. Targeted re-biopsies should be performed if cancer progression or higher-grade PCa is suspected on mp-MRI.

**Key Points:**

• *None of the patients with unsuspicious mp-MRI had a GS upgrade in re-biopsy and mp-MRI might replace serial biopsies in these cases*

• *More than 60% of patients with mp-MRI signs of tumor progression had subsequent Gleason score (GS) upgrades*

*• Targeted re-biopsies should be performed in case of higher GS cancer suspicion on mp-MRI*

## Introduction

Active surveillance (AS) is an increasingly applied therapy option for patients with low-risk prostate cancer (PCa) [1] to avoid overtreatment and thus spare men with presumably indolent disease potential complications and long-term effects [2]. According to current urological guidelines, monitoring of patients on AS is mainly based on serial prostate-specific antigen (PSA) testing and regular re-biopsies [3, 4] which might reveal histopathological tumor progression and induce definitive therapy, if needed. However, a large proportion of patients discontinue AS due to histological reclassification and noncompliance [5, 6]. The reason for histological reclassification in repeat biopsies is mainly the high rate of falsely too low Gleason score (GS) results in up to 50% of the cases in initial extended systematic transrectal ultrasound-guided biopsies (TRUS-GB), which used to be the standard method for selection of men eligible for AS and for monitoring [7–9]. Multiparametric magnetic resonance imaging (mp-MRI) and targeted MRI/US fusion-guided biopsy (FUS-GB) have been shown to substantially improve inclusion of patients in AS as they reduce the number of men with incorrectly diagnosed low-risk cancer that actually harbor clinically significant disease [10, 11]. Results from the ASIST trial revealed that baseline mp-MRI before confirmatory biopsy can significantly decrease the number of AS failures and of tumor progression to higher-grade cancer after a 2-year follow-up episode [12, 13].

Thus, mp-MRI and MR-guided biopsies have already been implemented into current guidelines to diminish the inclusion error [3, 4, 14]. Mp-MRI and MR-guided biopsies are also promising tools to optimize patient observation during AS and possibly minimize the overall number of re-biopsies. Published data already exists indicating that stability on mp-MRI is associated with histopathological stability [15–17] and that mp-MRI facilitates detection of tumor progression [18]. However, not all cases of histological tumor progression on AS could be identified on mp-MRI in these studies and many other authors do not recommend waiving standard systematic follow-up TRUS-GB in order not to miss clinically significant PCa (csPCa) development [19, 20]. Comparison of the existing published studies is complicated by different study protocols with differing inclusion criteria, biopsy methods and schedules, and especially various definitions of imaging signs of tumor progression. Recently, a task force of the European School of Oncology revealed the Prostate Cancer Radiological Estimation of Change in Sequential Evaluation (PRECISE) recommendations to guide clinical evaluation of individual serial prostate MRIs on AS and to allow standardized reporting of AS cohorts with defined radiological assessment of tumor progression using a 5-point Likert scale representing the likelihood of cancer progression [21]. Until today, data on serial mp-MRI using these standardized criteria in patients undergoing AS are lacking. Therefore, standardized mp-MRI-based monitoring of AS patients has not yet been implemented into current guidelines with exception of the UK National Institute for Clinical Excellence (NICE) guideline [14, 22].

Thus, the purpose of this study was to assess the ability of mp-MRI to exclude PCa progression in patients with low- and intermediate-risk PCa on AS and to compare rates of PCa upgrading using targeted FUS-GB vs traditional systematic TRUS-GB.

## Material and methods

### Study population and design

**Fig. 1:**
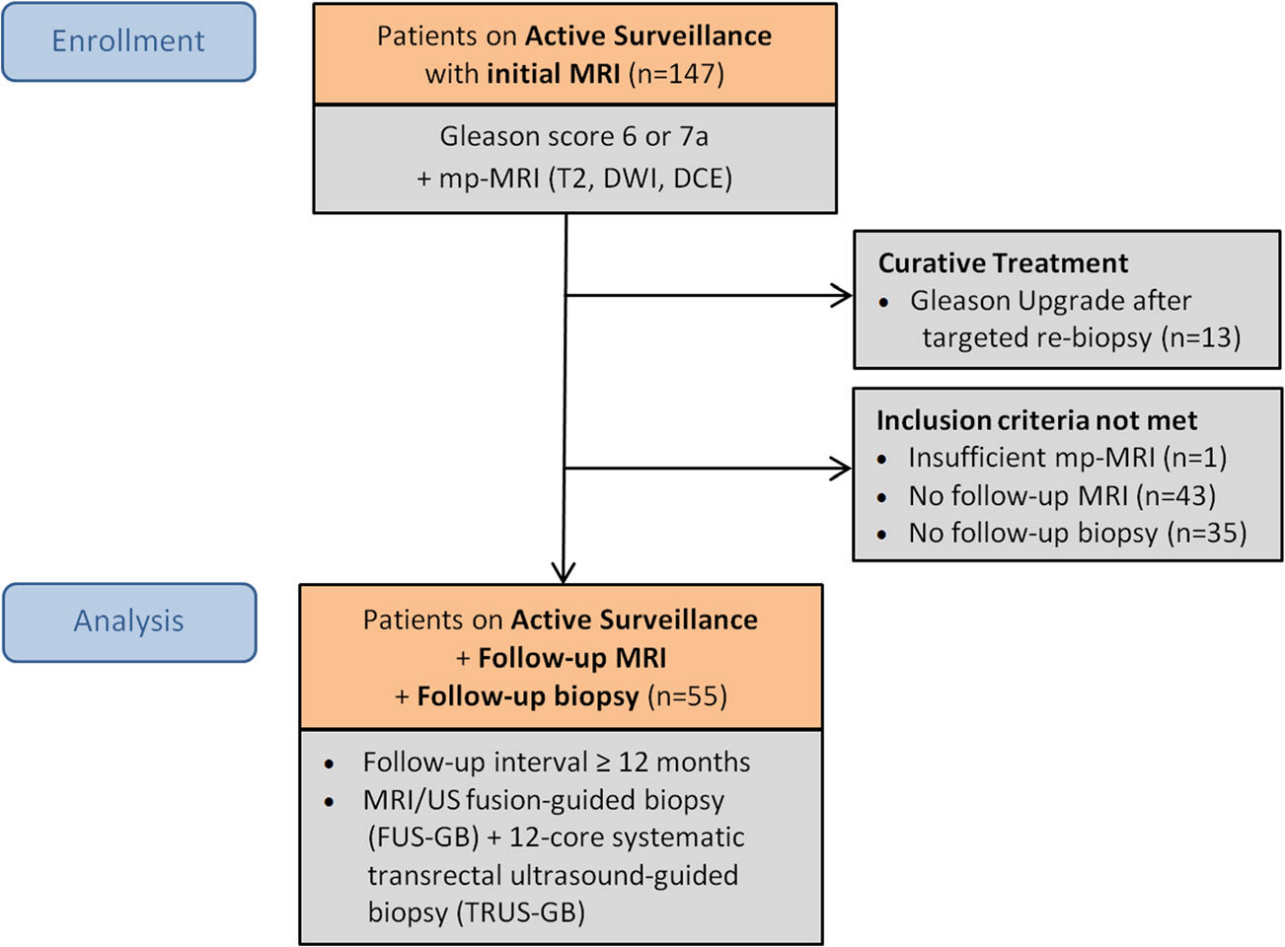
Study design and patient flow chart

In this retrospective single-center cohort study, 147 consecutive patients on AS with initially diagnosed PCa at the University of Duesseldorf or at an outside institution received mp-MRI at 3 Tesla between October 2011 and September 2017 at our hospital. Of these patients, 55 were finally included who received a follow-up mp-MRI after a minimum interval of 12 months (median 19 months; IQR 13–33 months) with subsequent targeted MRI/US fusion-guided follow-up biopsy (FUS-GB) and concurrent systematic TRUS-GB after a median interval of 41 days (IQR 32–67 days) (Fig. 1). Inclusion criteria were histologically verified PCa with a GS of 3 + 3 = 6 or 3 + 4 = 7a, i.e., low- or intermediate-risk by D’Amico histological criteria [23], initial mp-MRI, and follow-up mp-MRI (≥ 12 months) with subsequent targeted MR-guided plus systematic 12-core TRUS-guided biopsy. The follow-up mp-MRI and subsequent follow-up biopsies were set to occur 12 to 16 months after the initial, diagnostic biopsy according to in-house standard of AS monitoring. Confirmatory biopsies were regularly scheduled for AS patients independent of the mp-MRI results but imaging is used to detect and locate index lesions of potentially higher GS tumors. Information and PCa localization from the histopathological reports of the initial biopsies were used to correlate index lesions in the initial mp-MRI. If the mp-MRI did not show a PCa lesion, a representative lesion in the region of the pathologically positive results was determined. Exclusion criteria were insufficient MR imaging (*n* = 1), no follow-up mp-MRI (*n* = 43), or no follow-up biopsy (*n* = 35). Of all patients, 13 received definitive curative treatment after GS upgrade in the interim before follow-up mp-MRI and were also excluded. Earlier biopsies compared with standard AS regime were triggered by clinical factors (increase of PSA/PSAD and/or aggravating symptoms). Retrospective assessment of visible radiologic progression was performed, comparing the initial and follow-up mp-MRI. Finally, results of the image analyses were compared with the follow-up biopsies to evaluate the ability of mp-MRI to predict tumor progression. The study was approved by the local ethics committee with a waiver of written informed consent.

### Study endpoints

Primary endpoint of the study was the negative predictive value (NPV) of the follow-up mp-MRI to exclude tumor progression. Secondary endpoints were positive predictive value (PPV) (1), sensitivity (2), specificity (3), PCa upgrades including GS distribution (4), and benefit of TRUS-GB in addition to FUS-GB (5).

### Imaging

All mp-MRI scans were conducted on 3T MRI scanners (Magnetom TIM Trio, Prisma or Skyra; Siemens Healthcare GmbH) using either 18-channel phased-array surface coil combined with 32-channel spine coil or a 60-channel phased-array surface coil (anterior and posterior part integrate 30 elements each). MR imaging parameters were chosen according to international recommendations [24] and contained T2-weighted sequences in 3 planes (T2WI; turbo spin echo, TSE; axial: voxel size 0.5 × 0.5 × 3.0 mm; FOV 130 mm), diffusion-weighted imaging (DWI; EPI and RESOLVE; voxel size 0.9–1.4 × 0.9–1.4 × 3.0 mm; *b* values 0, 500, 1000 s/mm^2^ plus ≥ 1400 s/mm^2^), and dynamic contrast-enhanced imaging (DCE; T1 vibe; voxel size 0.8–1.5 × 0.8–1.5 × 3.0 mm, scan time 3 min, temporal resolution < 8 sec). Apparent diffusion coefficient (ADC) parameter maps were calculated by the scanner using the standard monoexponential model (including b0).

### Biopsy and histopathology

Patients received transrectal targeted follow-up FUS-GB and subsequent systematic 12-core TRUS-GB on a MRI/US fusion-guided biopsy system with elastic registration (Urostation, Koelis or UroNAV, Philips Healthcare) using an 18G fully automatic biopsy gun (Bard Medical). All biopsies were done by three experienced urologists (C.A., A.H., and D.M.) with 9, 8, and 5 years’ experience, respectively. Systematic TRUS-GB were conducted using a standardized biopsy plan which included lateral and midlobar cores at the base, middle, and apex of each prostate lobe. Gleason evaluation was performed by experienced uropathologists according to the recommendations of the International Society of Urological Pathology (ISUP) [25]. Histopathological cancer progression was defined as any increase in GS in any core in either TRUS-GB or FUS-GB.

### Data and image analysis

Image interpretation was done by two radiologists with 5 and 10 years’ experience according to PI-RADS v2.1 in consensus. Prostate volume was measured by software volumetric (DynaCAD, Philips Healthcare) and PSA density (PSAD) was calculated by dividing PSA blood levels by prostate volume.

The analysis of serial mp-MRIs to assess radiologic tumor progression was performed and reported according to the Prostate Cancer Radiological Estimation of Change in Sequential Evaluation (PRECISE) recommendations by the European School of Oncology using a 5-point Likert scale as measure of likelihood of tumor progression on AS [21]. Imaging signs of progression were thus defined as a significant increase in size of an index lesion, measured in 3 planes in T2WI or DWI and/or increase in conspicuity of features suspicious for PCa according to PI-RADS v2.1 [26] in any sequence and/or newly detectable, suspicious lesions. Scores of 4 and 5 were eventually defined as a positive mp-MRI for tumor progression. Scores of 1–3 were classified as stable imaging appearance including if the follow-up MRI continuously did not show a visible PCa lesion. Additionally, the results (radiological progression vs no progression) of the retrospective image analysis using the PRECISE criteria were compared with the results of the original mp-MRI reports in which similar decision criteria had been used. For the retrospective image analysis, the readers were blinded to the biopsy results and to the results of the original mp-MRI reports. Lesion volume was measured in axial and sagittal T2-weighted images (height × width × depths).

### Statistical analysis

Statistical analyses were performed using IBM SPSS® Statistics (version 21, IBM Deutschland GmbH). Data are expressed as mean ± SD and median + IQR. Patient demographic data were reported using descriptive statistics. Performance of the follow-up mp-MRI was assessed by determining PPV, NPV, sensitivity, and specificity relating to Gleason progression compared with subsequent biopsy. Exact binomial confidence limits were used for sensitivity, specificity, PPV, and NPV. Chi-square test was used to test for differences in proportions. Nonparametric data were tested with Mann-Whitney *U* test. Agreement between GS at TRUS-GB vs FUS-GB was evaluated with McNemar’s test of symmetry. Statistical significance was defined as *p* value < 0.05.

## Results

### Patients

Of the 55 enrolled patients, 42 had histologically proven PCa with a GS of 3 + 3 = 6 and 13 had PCa with a GS of 3 + 4 = 7. In 32 of all men, the previous, initial, diagnostic biopsy was a combination of targeted MR-guided biopsy plus systematic TRUS-GB, and in the remaining 23 men, the previous, diagnostic biopsy was a systematic TRUS-GB only. The intervals between initial and repeat mp-MRI did not differ significantly for the respective subgroups (*p* = 0.67 and *p* = 0.71, respectively). The clinical and demographic characteristics at the time of initial mp-MRI and follow-up mp-MRI are summarized in Table 1.

**Table 1:** Baseline characteristics at initial MRI and at follow-up MRI

		Patients with follow-up MRI and subsequent FUS-GB + TRUS-GB	
		Initial	Follow-up
Age (years)	mean ± SD	66 ± 7	68 ± 7
Prostate volume (ml)	median (IQR)	41 (30–54)	44 (30–60)
PSA (ng/ml)	median (IQR)	7.3 (4.9–9.7)	9.8 (5.7–13.9)
PSA density (ng/ml/ml)	median (IQR)	0.17 (0.11–0.27)	0.20 (0.15–0.30)

*PSA*, prostate-specific antigen; *FUS-GB*, targeted MRI/US fusion-guided biopsy; *TRUS-GB*, 12-core systematic transrectal ultrasound-guided biopsy; *SD*, standard deviation; *IQR*, interquartile range

### Prostate cancer detection and Gleason upgrade

**Fig. 2:**
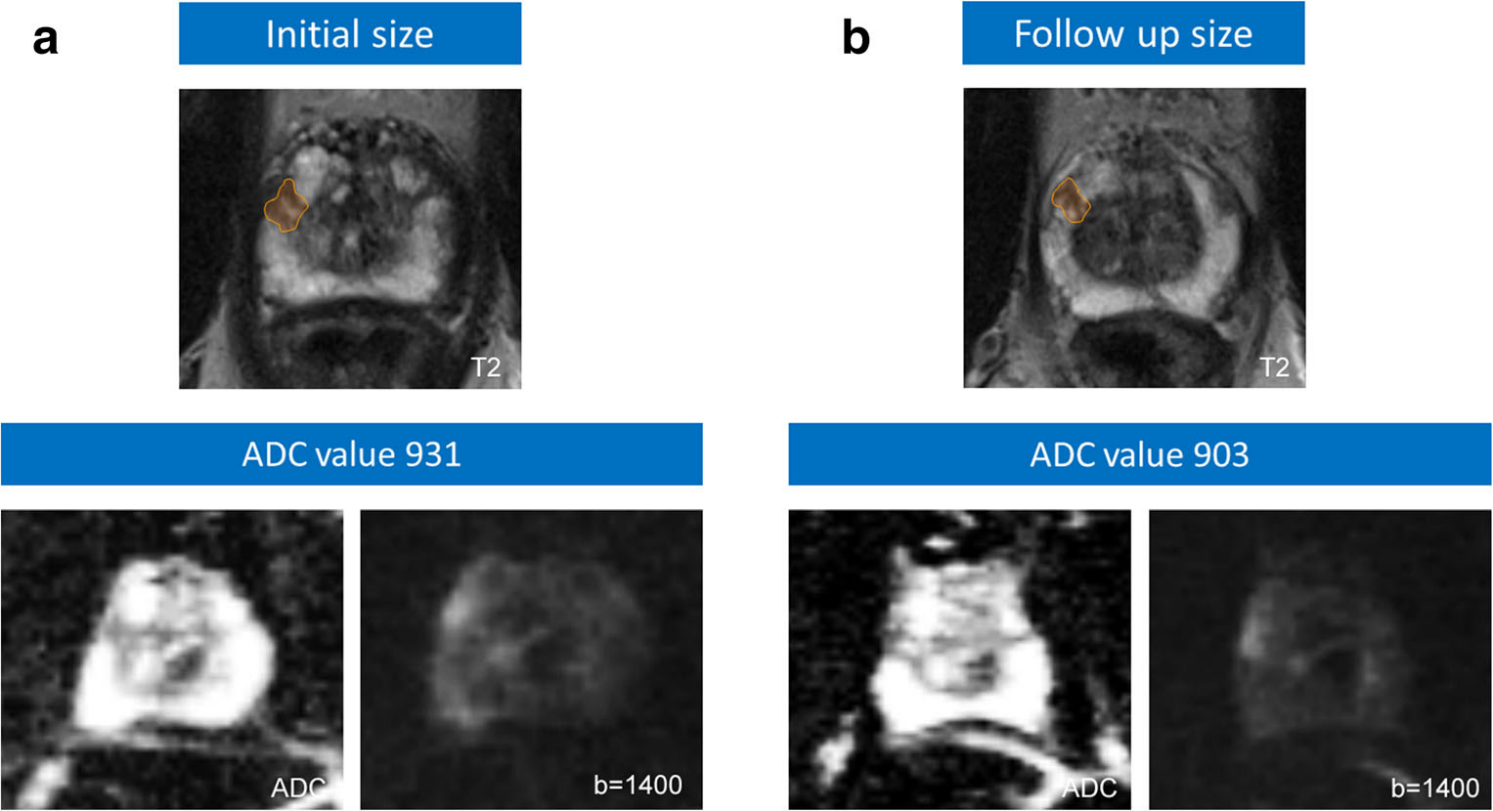
Example of a case with stable histopathology. **a** 69-year-old men with PSA value of 9.5 ng/ml and negative 12-core transrectal ultrasound-guided biopsy (TRUS-GB), but positive MR-guided biopsy (3 of 4 cores with Gleason score 3 + 3 = 6 in max. 80% of the core). **b** The follow-up-MRI 36 months later showed a stable MRI appearance in size and ADC value (PSA 10.5 ng/ml). MR-guided biopsy confirmed a persistent Gleason score 3 + 3 = 6 in max. 60% of the targeted biopsy cores (2 of 16 cores)

**Fig. 3:**
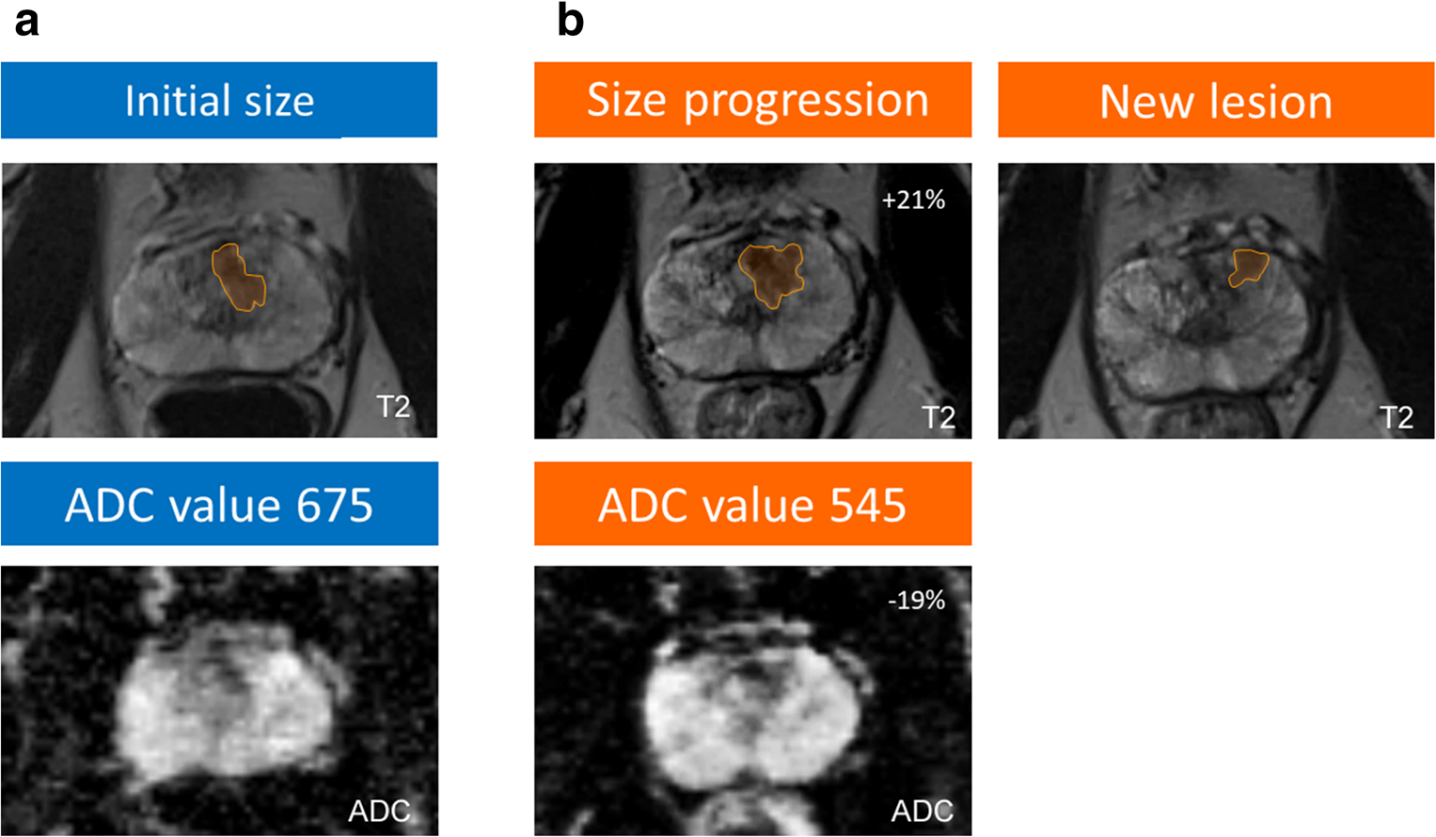
Example of a case with histological progression. **a** 67-year-old men with an initial PSA value of 10.3 ng/ml and a positive transrectal ultrasound-guided biopsy (TRUS-GB) (1 of 12 cores with Gleason score 3 + 3 = 6 in 5% of the core). **b** Follow-up-MRI 24 months later showed a MRI lesion progress in size, a significant ADC decrease of the lesion, and a new further lesion in the prostate apex (PSA increase to 12.7 ng/ml). MR-guided biopsy revealed a Gleason score upgrade to 3 + 4 = 7 in max. 40% of the targeted biopsy cores (6 of 17 cores)

Of 55 patients, 29 (53%) had histological tumor progression with a GS upgrade after confirmatory biopsy. Detailed Gleason distribution is shown in Table 2. Of 13 patients with initial GS of 3 + 4 = 7a, 5 (38%) had a GS upgrade. Of 42 patients with initial GS of 3 + 3 = 6, 24 (57%) had a GS upgrade. Of 23 patients with previous systematic TRUS-GB only, 14 (61%) had a GS upgrade compared with 15 (47%) patients with a GS upgrade in a group of 32 who had previous FUS-GB plus TRUS-GB. Differences in proportions of GS upgrades between the subgroups of initial GS 6 vs initial GS 7a and subgroups of previous TRUS-GB vs previous FUS-GB plus TRUS-GB, respectively, were not statistically significant (*p* = 0.24 and *p* = 0.31, respectively). The subgroup of patients with initial GS of 6 had more often received previous TRUS-GB only (45%) compared with the subgroup of patients with initial GS of 7a (31%); however, the difference was not statistically significant (*p* = 0.36). Figures 2 and 3 show examples of cases with stable histopathology and with histological progression, respectively. Lesion volumes of patients with GS upgrade were not significantly higher than lesion volumes of patients with stable GS (Mean ± SD (cm^3^) 0.76 ± 0.64 vs 0.72 ± 0.62, respectively; *p* = 0.40). The increase of the PSAD between the initial and the follow-up mp-MRI of patients with GS upgrade was not significantly higher than the PSAD increase of patients with stable GS (0.03 ng/ml/ml vs 0.03 ng/ml/ml; *p* = 0.80).

**Table 2:** Gleason score distribution after follow-up biopsy of all patients and of subgroups depending on initial GS score and previous biopsy method

	Highest GS after follow-up biopsy (%)					
Initial GS of all patients		3 + 3 = 6	3 + 4 = 7a	4 + 3 = 7b	4 + 4 = 8	4 + 5 = 9
	3 + 3 = 6	18 (33)	10 (18)	8 (15)	3 (5)	3 (5)
	3 + 4 = 7a	-	8 (15)	2 (4)	2 (4)	1 (2)
	Highest GS after follow-up Biopsy in patients w previous TRUS-GB* (%)					
Initial GS in patients w previous TRUS-GB*		3 + 3 = 6	3 + 4 = 7a	4 + 3 = 7b	4 + 4 = 8	4 + 5 = 9
	3 + 3 = 6	7 (30)	4 (17)	5 (22)	0	3 (13)
	3 + 4 = 7a	-	2 (9)	1 (4)	0	1 (4)
	Highest GS after follow-up Biopsy in patients w previous FUS-GB** (%)					
Initial GS in patients w previous FUS-GB + TRUS-GB**		3 + 3 = 6	3 + 4 = 7a	4 + 3 = 7b	4 + 4 = 8	4 + 5 = 9
	3 + 3 = 6	11 (34)	6 (19)	3 (9)	3 (9)	0
	3 + 4 = 7a	-	6 (19)	1 (3)	2 (6)	0

*GS*, Gleason score; *FUS-GB*, targeted MRI/US fusion-guided biopsy; *TRUS-GB*, 12-core systematic transrectal ultrasound-guided biopsy

*Patients that had initially only systematic TRUS-GB at the time when they were included in AS

**Patients that had initially combined FUS-GB + TRUS-GB at the time when they were included in AS

### Performance of mp-MRI in detection of histological tumor progression on AS

Overall, 44 men (80%) demonstrated a progression on mp-MRI, of whom 29 had a GS upgrade in the following biopsy. The remaining 11 men (20%) had no signs of progression on mp-MRI and none of them had histological tumor progression. The overall sensitivity and specificity of mp-MRI for histological progression were 100% (CI 0.88–1) and 42% (CI 0.26–0.61), respectively. The NPV and PPV were 100% (CI 0.74–1) and 66% (CI 0.51–0.78), respectively. Overall accuracy was 73% (CI 0.59–0.84). Table 3 illustrates the performance of mp-MRI in predicting GS progression in all patients and in subgroups depending on initial GS and prior biopsy method. Comparison of the results (progression vs no progression) of the follow-up mp-MRI and the original mp-MRI reports was in accordance in all cases.

**Table 3:** Expanded 2 × 2 table relating patients who progressed by mp-MRI or Gleason grade—analysis of all patients and subgroups depending on initial GS score and previous biopsy method

All patients (%)			
Gleason score upgrade	MRI progression	No MRI progression	Total
Yes	29 (53)	0	29 (53)
No	15 (27)	11 (20)	26 (47)
Total	44 (80)	11 (20)	55 (100)
Patients with initial GS of 3 + 3 = 6 (%)			
Gleason score upgrade	MRI progression	No MRI progression	Total
Yes	24 (57)	0	24 (57)
No	9 (21)	9 (21)	18 (43)
Total	33 (79)	9 (21)	42 (100)
Patients with initial GS of 3 + 4 = 7a (%)			
Gleason score upgrade	MRI progression	No MRI progression	Total
Yes	5 (38)	0	5 (38)
No	6 (46)	2 (15)	8 (62)
Total	11 (85)	2 (15)	13 (100)
Patients with previous TRUS-GB* (%)			
Gleason score upgrade	MRI progression	No MRI progression	Total
Yes	14 (61)	0	14 (61)
No	4 (17)	5 (22)	9 (39)
Total	18 (78)	5 (22)	23 (100)
Patients with previous FUS-GB + TRUS-GB** (%)			
Gleason score upgrade	MRI progression	No MRI progression	Total
Yes	15 (47)	0	15 (47)
No	11 (34)	6 (19)	17 (53)
Total	26 (81)	6 (19)	32 (100)

*GS*, Gleason score; *FUS-GB*, targeted MRI/US fusion-guided biopsy; *TRUS-GB*, 12-core systematic transrectal ultrasound-guided biopsy

*Patients that had initially only systematic TRUS-GB at the time when they were included in AS

**Patients that had initially combined FUS-GB + TRUS-GB at the time when they were included in AS

### Comparison of targeted and systematic biopsies

FUS-GB detected PCa in 46 of 55 (84%) individuals and lead to a GS upgrade in 28 cases (51%) (Table 4). TRUS-GB alone detected significantly fewer PCa with 36 cases (65%; *p* = 0.007) and lead to significantly fewer GS upgrades with 12 cases (22%; *p* < 0.001). TRUS-GB detected one GS 4 + 5 = 9 PCa that was not detected in FUS-GB. The combination of both techniques detected 47 PCa (85%) and let to a GS upgrade in 29 cases (53%). The differences in PCa detection and GS upgrade between FUS-GB and the combined approach was not statistically significant (Table 5).

**Table 4:** Prostate cancer detection and Gleason score distribution in targeted MRI/US fusion-guided biopsy (FUS-GB) and 12-core systematic transrectal ultrasound-guided biopsy (TRUS-GB) after follow-up MRI

	FUS-GB							
TRUS-GB	GS	Neg	3 + 3	3 + 4	4 + 3	4 + 4	4 + 5	Total
	Neg	*8*	2	2	4	2	1	19
	3 + 3	0	*10*	7	2	0	0	19
	3 + 4	0	0	*7*	2	0	1	10
	4 + 3	0	0	0	*2*	1	0	3
	4 + 4	0	0	1	1	*0*	1	3
	4 + 5	1	0	0	0	0	*0*	1
	Total	9	12	17	11	3	3	55

*GS*, Gleason score; *FUS-GB*, targeted MRI/US fusion-guided biopsy; *TRUS-GB*, 12-core systematic transrectal ultrasound-guided biopsy

**Table 5:** Comparison of prostate cancer detection rates and number of GS upgrades in targeted MRI/US fusion-guided biopsy (FUS-GB), 12-core systematic transrectal ultrasound-guided biopsy (TRUS-GB), and combined approach

		*N*	Detection rates (%)	*p* value^a^
FUS-GB vs TRUS-GB	Any PCa detection	46 vs 36	84 vs 65	*0.007*
FUS-GB vs combination		46 vs 47	84 vs 85	0.3
TRUS-GB vs combination		36 vs 47	65 vs 85	*< 0.001*
Gleason upgrade from initial GS				
FUS-GB vs TRUS-GB	Gleason upgrade	28 vs 12	97 vs 41	*< 0.001*
FUS-GB vs combination		28 vs 29	97 vs 100	0.3
TRUS-GB vs combination		12 vs 29	41 vs 100	*< 0.001*

*FUS-GB*, targeted MRI/US fusion-guided biopsy; *TRUS-GB*, 12-core systematic transrectal ultrasound-guided biopsy; *PCa*, prostate cancer; *GS*, Gleason score

^a^McNemar test was used to test for statistical significance; italicized table entries indicate statistically significant difference

## Discussion

The optimal follow-up strategy for men on AS is still a matter of debate as traditionally performed serial biopsies in combination with PSA testing can entail unnecessary complications, aggravated by the increasing problem of multidrug-resistant bacteria, and limited by poor compliance [5, 6]. Mp-MRI of the prostate is already recommended and commonly applied to select appropriate candidates for AS and target suspicious lesions in initial or confirmatory biopsies [27, 28]. In this study, we demonstrate that mp-MRI is also an excellent monitoring tool for follow-up of patients on AS. We revealed a high NPV and sensitivity for follow-up mp-MRI in detecting histological tumor progression in men with known PCa on AS using subsequent targeted FUS-GB and concurrent systematic TRUS-GB as reference standard. Thus, if only patients with signs of mp-MRI progression had undergone follow-up biopsy, 11 patients could have safely avoided repeat biopsy. Mp-MRI seems to be a valuable monitoring tool in patients undergoing AS reducing the number of invasive procedures, increasing patient comfort and compliance.

The NPV in our study was even higher than the results from Walton Diaz et al [15] who also reported a high NPV of 80% (95% CI 65–91%) for mp-MRI in a cohort of 58 men on AS and from Felker et al [29] who revealed a NPV of 70%. A possible explanation is that we might have used a lower, more sensitive threshold to diagnose radiological tumor progression, which would also explain the lower specificity in our study. However, this approach allowed us to confidently exclude tumor progression and safely avoided repeat biopsy without missing a single cancer progression. Our NPV was also higher than values reported in the PROMIS trial in which Ahmed et al [7] revealed a NPV of 76% for detection of clinically significant PCa (csPCa) in mp-MRI in biopsy-naïve men. The higher the NPV for the follow-up method, the less likely it is to miss significant cancer progression and the safer it is for the patients on AS to waive follow-up biopsy. Risks and benefits of follow-up biopsies have to be thoroughly weighed considering the overall low mortality of clinically localized PCa [30]. Even if mp-MRI may miss tumor progression at some point, serial MRIs within the regime of AS might allow detection of progression in a further follow-up examination before clinically significant disease occurs.

In contrast, some previous studies reported a considerably worse performance of mp-MRI in predicting cancer progression on AS [19]. Ma et al even revealed a lower sensitivity for MRI-targeted biopsy than for random systematic biopsy in csPCa detection in an AS cohort [31]. Partly, the differing results can be explained by the vast heterogeneity of the used AS protocols with various follow-up methods, schedules, and different inclusion criteria. In the original Epstein criteria, AS was suggested only for patients with small GS 3 + 3 = 6 PCa [32], but over the years, many programs have extended AS to those with more extensive, bigger lesions and even to low-volume GS 3 + 4 = 7a tumors [33], i.e., low- or intermediate-risk by D’Amico histological criteria [23].

In our study, no significant difference in progression between baseline GS 6 and GS 7a tumors was present. Our subgroup analyses also excluded initial biopsy method prior to study entry or lengths of the intervals between the mp-MRI studies as significant reasons for this difference.

Another very important reason for the differing performances of mp-MRI in predicting tumor progression on AS reported in the literature is the lack of standardized imaging criteria to determine mp-MRI tumor progression. It has not yet been sufficiently investigated which increase in size constitutes tumor progression, which is the most reliable method to measure tumor size and which other parameters could play a role in detecting tumor progression, for example, the decrease of ADC values [19]. The excellent, comprehensive, and recently updated PI-RADS v2.1 handbook states that recommendations do not address the use of mp-MRI for detection of progression during AS [26]. We used the recently revealed Prostate Cancer Radiological Estimation of Change in Sequential Evaluation (PRECISE) recommendations that hopefully facilitate comparison of AS cohorts through standardized definition of radiological assessment of tumor progression [21]. However, the exact imaging criteria of tumor progression and distinction between significant change, measurement error, and natural fluctuations in tumor appearance have yet to be investigated. Until then, even when using the standardized PRECISE recommendations, the criteria are subjective to a certain extent, so that sensitivity and related parameters can vary between different readers. Another explanation that may have contributed to the high NPV in our study was the limited number of patients.

In the context of AS, another important task of prostate mp-MRI and MR-guided biopsies is the decreasing of the sampling error for initial selection of appropriate candidates. In our study, 13 patients received definitive, curatively intended treatment before the follow-up mp-MRI was performed due to GS upgrade after confirmatory targeted biopsy using the information of the initial mp-MRI. These patients were not part of the final analysis. However, there may still be an inclusion or sampling error since not all of the finally analyzed patients received MR-targeted FUS-GB when they were included in our study. Consequently, a definite differentiation between inclusion error and tumor progression on AS is not possible. However, as many outside patients just receive initial TRUS-GB before inclusion in AS, this problem might not mitigate until targeted FUS-GB is more commonly used.

The overall histopathological tumor progression on AS in our study was high compared with values in the current literature [12, 13, 28]. A possible reason for that might be a higher rate of underdiagnosed higher-grade cancers in the initial, diagnosing biopsies.

Another important observation in our study is that targeted confirmatory FUS-GB lead to significantly more GS upgrades compared with confirmatory systematic TRUS-GB. The combined approach of targeted and systematic biopsy revealed more GS upgrades than FUS-GB alone, though the difference was not statistically significant, which is in line with the findings by other studies [10] and supports the use of mp-MRI-targeted biopsy in follow-up examinations of men on AS.

In our evaluation, the increase of PSAD between the time points of the two mp-MRIs was not statistically different for patients with and without GS upgrade even if previous studies proposed PSAD as independent risk factor for PCa. It has to be mentioned though, that in our study, the median PSAD at the initial mp-MRI is already above 0.15 ng/ml/ml, which in many studies is suggested as decisive threshold [34].

Our study has limitations. In addition to its retrospective nature, the study cohort was limited in number and still heterogeneous with some patients having received only TRUS-GB prior to our study as initial AS inclusion method, as discussed above. However, all patients received combined confirmatory targeted FUS-GB and TRUS-GB in our study protocol. We did not use radical prostatectomy as final reference standard. However, it has been shown that FUS-GB and concurrent TRUS-GB can reliably detect PCa when compared with prostatectomy. The study focused on GS upgrade and did not address number of positive cores, percentage of core involvement, or progression in size; tumor progression might be present in cases with mp-MRI progression, but without GS upgrade in subsequent biopsy.

In conclusion, none of the patients with unsuspicious mp-MRI had a GS upgrade in re-biopsy giving rise to the idea that mp-MRI might allow waiving serial follow-up biopsies on AS under the precondition of stable clinical status. Targeted re-biopsies should be performed if higher GS cancer is suspected on mp-MRI. Further prospective studies are warranted to investigate the performance of mp-MRI in follow-up of AS to ultimately improve safety and compliance of AS with less invasive methods.

## Abbreviations

ACR: American College of Radiology
ADC: Apparent diffusion coefficient
AS: Active surveillance
csPCa: Clinically significant prostate cancer
DCE: Dynamic contrast-enhanced imaging
DWI: Diffusion-weighted imaging
FUS-GB: MRI/US fusion-guided biopsy
GS: Gleason score
IQ: Image quality
IQR: Interquartile range
mp-MRI: Multiparametric magnetic resonance imaging
PCa: Prostate cancer
PI-RADS: Prostate Imaging Reporting and Data System, version 2.1
PSA: Prostate-specific antigen
T2WI: T2-weighted imaging
TRUS-GB: Transrectal ultrasound-guided biopsy
